# Are We Missing Hypothyroidism among Children with Sickle Cell Anaemia?

**DOI:** 10.4314/ejhs.v33i6.6

**Published:** 2023-11

**Authors:** SO Akodu, AF Adekanmbi, TA Ogunlesi, MB Fetuga

**Affiliations:** 1 Department of Paediatrics, Olabisi Onabanjo University Teaching Hospital, Sagamu, Ogun, Nigeria

**Keywords:** Sickle cell anaemia, Thyroid hormones, Thyroid Stimulating Hormone, Hypothyroidism, Children, Nigeria

## Abstract

**Background:**

Children with sickle cell anaemia have been reported to have potential risk of hypothyroidism from chronic blood transfusions and probable thyroid tissue ischaemia. However, few studies on hypothyroidism status of children with sickle cell anaemia in Nigeria are available. The objective of this study was to determine the prevalence of hypothyroidism among children with sickle cell anaemia.

**Methods:**

A cross sectional study that assayed the thyroid hormones and thyroid stimulating hormone (TSH) of 71 children with sickle cell anaemia was conducted at Olabisi Onabanjo University Teaching Hospital Sagamu. Using age appropriate hormonal reference values, the subjects were classified into subclinical, primary and secondary hypothyroidism.

**Results:**

The mean serum TSH, Free T3, and Free T4 were comparable irrespective of age category (p > 0.05). No subject was identified to have low TSH value while 7.0% had high TSH value. Low free T3 was identified in 1.4% and 8.5% had high free T3 values. Low free T3 and free T4 were seen in 11.3% each of the subjects. The overall prevalence of primary, secondary and subclinical hypothyroidism was 0%, 0% and 4.2%, respectively.

**Conclusion:**

Sub-clinical hypothyroidism does occur in Nigerian children with sickle cell anaemia. Routine screening for hypothyroidism is advocated in all children with sickle cell anaemia.

## Introduction

Sickle cell anaemia (SCA) is the most common inherited chronic haemolytic anaemia caused by a structurally abnormal variant of haemoglobin that is inherited in an autosomal recessive fashion (1,2). The disease is characterized by polimerization of the haemoglobin S (HbS) with deformation of red blood cells into sickle shapes that results in obstruction to blood flow, tissue hypoxia and organ failure (3,4). The condition manifests with acute and chronic vaso-occlusion and multiple organ dysfunction resulting from repeated microcirculation occlusion by the sickle red cells with recurrent episodes of ischaemia-reperfusion injury (4,5,6,7).

Poor growth and pubertal delay are the most frequent disorders observed in children and adolescents with SCA (8). Children with SCA have been reported to experience various endocrine dysfunctions and this may very well be responsible for the poor growth and delayed puberty associated with the disease (9). The burden of endocrine and metabolic disorders in children and adolescents with SCA varies in different populations depending on the literacy rate, socioeconomic status, and access to appropriate treatment (8).

The thyroid gland is an endocrine gland which produces thyroid hormones that primarily influence metabolic rate. The metabolic effects of thyroid hormones have been directly linked to Reactive Oxygen Species (ROS) production and oxidative stress (10); both Reactive Oxygen Species (ROS) production and oxidative stress are often experienced by patients with sickle cell disease (11,12). Both hyperthyroidism and hypothyroidism have been shown to be associated with ROS (13,14).

The aetiology of thyroid dysfunction in children with SCA is not clear; however, mechanisms implicated in the development of hypothyroidism in children with SCA include iron overload as a result of chronic haemolysis and frequent blood transfusions, chronic anaemia and microcirculation occlusion by the sickled red cells with recurrent episodes of ischaemia-reperfusion injury to thyroid tissue (9). Investigators have also hypothesized that thyroid dysfunction in children with SCA may be caused by damage to thyroid tissue by inflammatory mediators (9). Increased duration of the disease with requirement of transfusion therapy of more than eight transfusions per year has been defined as predictors of iron overload (9).

Hypothyroidism may affect the brain and physical development leading to growth retardation and impaired bone maturation in children with SCA (9). Despite these consequences of hypothyroidism in children with SCA, routine screening of children for hypothyroidism is not currently practiced in Nigeria. Existing studies about thyroid abnormalities among children with sickle cell

anaemia to required to assist the clinicians in early detection, diagnosis, and prevention through early intervention are few locally. Therefore, there is a clear need to know the thyroid hormones status of children with sickle cell anaemia in a Nigerian setting. The aim of this study is to determine the thyroid hormone status of children with sickle cell anaemia in steady state in Ogun State, Southwest Nigeria.

## Methods and Subjects

**Study location and study design**: The present study was a prospective, cross-sectional one conducted in the Paediatric Haematology Outpatient Clinic of the Department of Paediatrics, Olabisi Onabanjo University Teaching Hospital (OOUTH), Sagamu, Ogun State in Southwest Nigeria between 1^st^ August 2019 and 28^th^ February 2020.

**Study population**: The study population comprised all children with SCA aged 5 – 15 years in steady state, attending the Paediatric Haematology Outpatient Clinic of the Department of Paediatrics (OOUTH) during the study period. All children with SCA in steady state aged 5 – 15 years were consecutively recruited till the calculated sample size was achieved. A child with SCA was said to be in steady state in the absence of any sickle cell crises in the preceding four weeks, no recent drop in haemoglobin level and absence of any symptoms or signs attributable to an acute illness (15). SCA subjects in steady state were included in this study. On the other hand, children with recent history of blood transfusion and chronic conditions such as hepatic or renal insufficiency or any known endocrine disease were excluded. Also excluded from the study were children receiving medications such as rexinoids, dopamine agonists and iodine supplements which have variable effects on thyroid functions.

**Clinical data collection**: Detailed history taking as well as general and physical examinations were carried out. A single observer performed the physical examination of all the study subjects. Details were documented in a pre-designed questionnaire.

**Procedure for thyroid hormone estimation**: Three millilitres of blood were drawn from all study subjects between 8:00am –10:00am from a convenient peripheral vein and transferred into plain vacutainer tubes. The vacutainer tubes were labelled and were transported to the Chemical Pathology Laboratory of Olabisi Onabanjo University Teaching Hospital for analysis within one hour of collection. The fresh blood samples were centrifuged at 3,500 revolutions per minute for 15 minutes using the Uniscope Laboratory Centrifuge, Model SM 112 (Surgifriend Medicals, England). Serum was separated after centrifuge, and was analysed for serum thyroid stimulating hormone (TSH), free triiodothyronine (free T3) and free thyroxine (free T4) levels by enzyme-linked immunosorbent assay (ELIZA) using Cobas 6000 analyzer by Roche Diagnostics USA. The manufacturer's guidelines were followed.

Table 1 shows the standard paediatric reference range (16) for all the TSH, free T3 and free T4 measurements compared with the results obtained. They were subsequently used to categorize the patients. Subjects with values below, within or above the reference range were classified into low, normal and high TSH/freeT3/free T4, respectively. Children were categorized into three groups according their serum TSH, free T3 and free T4 levels based on the criteria previously reported in the literature (9). Primary hypothyroidism: elevated serum TSH level in combination with low serum free T4 level (9).

**Table 1:** Thyroid function test normal reference range values (9)

Test	Age (years)	Reference Range
**Serum TSH (µIU/ml)**	1 – 5	0.7 – 6.6
	6 – 10	0.8 – 6.0
	11 – 18	0.6 – 5.8
**Serum Free T3 (pg/ml)**	1 – 5	2.73 – 4.9
	6 – 10	2.73 4.69
	11 – 18	2.57 – 4.
**Serum Free T4 (ng/ml)**	1 – 5	0.8 – 1.8
	6 – 10	1.0 – 2.1

	11 – 18	0.8 – 1.9

**Secondary hypothyroidism**: Low serum TSH level in combination with either low, normal, or slightly elevated serum free T4 level (9). Subclinical hypothyroidism: elevated serum TSH level and normal serum free T4 level (9).

**Procedure for Packed Cell Volume (PCV) estimation**: Two millilitres of blood were drawn from a convenient peripheral vein and transferred into EDTA containing tubes for determination of Packed Cell Volume (PCV) using microhaematocrit method.

**Nutritional status**: Each child's weight was measured to the nearest one decimal place (0.1 kg) using a calibrated weighing scale (Seca®). The length/height was measured to the nearest 0.1 cm using infantometer or stadiometer (Seca®) depending on whether the children can stand or not. Body Mass Index (BMI) was obtained using the formula: weight divided by the square of height in meters (kg/m^2^). Weight-for-age, height-for-age, weight-for-height and BMI z-scores were calculated based on the WHO growth standards of 2006. The study subjects were classified into different types of under nutrition using their weight-for-age, height-for-age, weight-for-height and BMI z-scores. Under-nutrition was categorized using the WHO classification as follows: A weight-for-age, height-for-age, weight-for-height and BMI z score ≥-2 was classified as no malnutrition, while Weight-for-age, height-for-age, weight-for-height and BMI z score <-2 was classified as wasting/ underweight/stunting/thinness, respectively (17). Packed Cells Volume below 20% was regarded as severe anaemia (18).

**Sample size**: The sample size was calculated using the formula (19): (Z_α/2_)^2^(SD)^2^/d^2^, where Z_α/2_ is the critical value of normal distribution at 95% confidence level (1.960), SD is the standard deviation of free thyroxine (free T4) values from a previous study by Hagag *et al.* (20), which is 0.174 ng/dl and d is the desired margin of error of 5%. Substituting these figures into the formula, the calculated sample size was 47. Additional 50% (24 subjects) of the calculated sample size was recruited to accommodate possible attritions and possibly to increase the power of the study. This brought the calculated minimum study sample size to 71. Therefore, 71 children with SCA were conveniently recruited, except those who did not give consent.

**Statistical analysis**: The collected data were entered in Microsoft Excel and Statistical analyses were conducted using the Statistical Package for the Social Sciences (SPSS) version 17.0. Means of normally distributed data were compared using the Student's t-test. Categorical variables were compared using Pearson's Chi-square test as indicated. Correlations between variables with normal and non-normal distribution were performed using Pearson's and Spearman's correlation tests, respectively. *P* values < 0.05 were considered significant.

**Ethical considerations**: Ethical approval of the study was granted by the Health Research Ethics Committee of the Olabisi Onabanjo University Teaching Hospital (HREC/257/2019).

## Results

**Characteristics of study participants**: We enrolled 71 children with SCA aged five to 15 years. The mean age (SD) was 9.37 (±3.29) years. Close to two-thirds (60.6%) of the study subjects were within the age category 5 – 10 years. Slightly above half of the study subjects were males. As regards the nutritional status, the rate of wasting, underweight, stunting, and thinness among the study subjects were 8.5%, 0.0%, 0.0% and 0.0%, respectively. Ten (14.1%) of the study subjects had severe anaemia. The characteristics of the study subjects are as shown in Table 2.

**Table 2:** Characteristics of study participants

Characteristics		Frequency	Percnet
Mean (SD) age at recruitment (years)	9.37 (±3.29)		
Age Group	5 – 10 years	43	60.6
	>10 years	28	39.4
Gender	Male	36	50.7
	Female	35	49.3
Nutritional Status	Wasting	6	8.5
	No Wasting	65	91.5
Severe Anaemia	Yes	10	14.1
	No	61	85.9

**Laboratory and anthropometric findings of study participants**: The mean laboratory and anthropometric values of the study subjects are shown in Table 3. The mean Packed Cells Volume, Serum TSH, Serum Free T3, and Serum Free T4 were comparable irrespective of age category (p > 0.05). The mean anthropometric values (weight-for-age, height-for-age, weight-for-height and BMI) were significantly higher among study subjects >10 years compared with their counterpart aged 5 – 10 years (p < 0.05).

**Table 3:** Mean Laboratory and anthropometric findings of study participants

Variables	5 – 10 years	>10 years	ALL	p-value
Packed Cells Volume (%)	23.50 (4.54)	23.74 (4.23)	23.58 (4.38)	0.818
Serum TSH (µIU/ml)	3.34 (1.71)	2.82 (1.11)	3.14 (1.52)	0.157
Serum Free T3 (pg/ml)	3.45 (0.68)	3.17 (0.81)	3.34 (0.74)	0.118
Serum Free T4 (ng/dl)	1.21 (0.55)	1.04 (0.47)	1.13 (0.53)	0.190
Weight (Kg)	21.11 (4.68)	35.89 (6.58)	26.94 (9.10)	<0.0001
Height (m)	120.09 (10.96)	146.93 (10.03)	130.68 (16.89)	<0.0001
Weight-for-Height (Kg/m)	0.17 (0.03)	0.24 (0,24)	0.20 (0.04)	<0.0001
Body Mass Index (Kg/m^2^)	14.44 (1.33)	16.53 (2.01)	15.26 (1.91)	<0.0001

**Thyroid hormonal parameters abnormalities in the studied population**: In Table 4, the mean values of thyroid hormonal parameters among study subjects are shown. No subject was identified to have low TSH value while 5.6% of the study subjects had high TSH values. Only one subject had low free T3 value while 8.5% of the study subjects had high free T3 values; 11.3% of the study subjects had either low free T4 values or high free T4 values.

**Table 4:** Thyroid hormonal parameters abnormalities in the studied population

Thyroid Hormone		5 – 10 years n (%)	>10 years n (%)	ALL n (%)	p-value
Serum TSH (µIU/ml)					0.543
	Low	0 (0.0)	0 (0.0)	0 (0.0)	
	Normal	40 (93.0)	27 (96.4)	67 (94.4)	
	High	3 (7.0)	1 (3.6)	4 (5.6)	
Serum Free T3 (pg/ml)					0.384
	Low	0 (0.0)	1 (3.6)	1 (1.4)	
	Normal	40 (93.0)	24 (85.7)	64 (90.1)	
	High	3 (7.0)	3 (10.7)	6 (8.5)	
Serum Free T4 (ng/dl)					0.810
	Low	4 (9.3)	4 (14.3)	8 (11.3)	
	Normal	34 (79.1)	21 (75.0)	65 (91.4)	
	High	5 (11.6)	3 (10.7)	8 (11.3)	

**Prevalence of hypothyroidism in the studied population**: The overall prevalence of primary, secondary and sub-clinical hypothyroidism was 0%, 0% and 4.2%, respectively. There was no child with primary or secondary hypothyroidism in this study. However, three subjects (7.0%) aged 5-10 years and none (0.0%) aged >10 years enrolled were found to have sub-clinical hypothyroidism. The prevalence of sub-clinical hypothyroidism was higher among subjects with age category 5-10 years, males, with wasting and severe anaemia (Table 5).

**Table 5:** Prevalence of hypothyroidism in the studied population

Variables		Sub-Clinical Hypothyroidism n/(%)	Primary Hypothyroidism n/(%)	Secondary Hypothyroidism n/(%)	p-value
Age Group					0.298
	5 – 10 years	3 (7.0)	0 (0.0)	0 (0.0)	
	>10 years	0 (0.0)	0 (0.0)	0 (0.0)	
Gender					0.191
	Male	3 (8.3)	0 (0.0)	0 (0.0)	
	Female	0 (0.0)	0 (0.0)	0 (0.0)	
Nutritional Status					0.160
	Wasting	1 (16.7)	0 (0.0)	0 (0.0)	
	No Wasting	2 (3.1)	0 (0.0)	0 (0.0)	
Severe Anaemia					0.352
	Yes	1 (10.0)	0 (0.0)	0 (0.0)	
	No	2 (3.3)	0 (0.0)	0 (0.0)	

## Discussion

Thyroid hormones are required the for maintenance of the body basic metabolic and neurophysiologic functions, and it is regulated through the thyroid-pituitary pathway (21). TSH, FT4 and FT3 are the hormones responsible for these body functions and their levels are regulated by a complex negative feedback mechanism. Nigeria, like many African countries, has made attempts to start the newborn screening for congenital hypothyroidism but failed (22).

In the index study, there was no significant difference in the mean values of all the serum thyroid hormones irrespective of age category. The mean TSH value of 3.14 µIU/ml was higher than 3.01 µIU/ml reported by Kaudha *et al.* (9) among Ugandan children with SCA aged six months to 17 years but lower than 4.61 µIU/ml reported by Hagag *et al.* (20) among Egyptian children with SCA aged 11 – 14 years. The observed difference in mean TSH values maybe the effect of the disparity in the age group of recruited subjects across study, as aging has been reported to be associated with increased serum TSH concentration (23).

The mean thyroxine values reported in the present study was higher than 0.91 ng/dl reported by Hagag *et al.* (20) among Egyptian children with SCA but lower than 1.36 ng/dl by Kaudha *et al.* (9) among Ugandan children with SCA aged six months to 17 years. The observed difference between both studies may also be due to the disparity in environmental factors across the studies which would be very difficult to compare. It has been reported that environmental factors such as lifestyle, diet and exercise influence thyroid hormones levels (24).

The T3 is the key metabolic regulator controlling the short-term and long-term energy needs of the body (25). The mean T3 values of the children with SCA recruited for this study was higher than the mean value of 2.61pg/ml reported by Hagag *et al.* (20) among Egyptian children with SCA. The higher mean value of T3 among our study subjects may be the effect of higher basal metabolic requirement, tissue resistance to thyroid hormones and/or selective resistance in the pituitary gland in the subjects. The latter is outside the scope of the current study.

The overall prevalence of sub-clinical hypothyroidism reported in the present study is within the range of 2 – 6% reported by previous researchers (8,26,27). On the contrary, this reported prevalence rate was lower to what Gomes *et al.* (28) reported amongst thirty Brazilian children with sickle cell anaemia aged > 10 years. The observed difference is possibly an effect of the characteristics of the population studied. Small sample size such as was the case in the study by Gomes *et al.* (28) is known to produce exaggerated prevalence rates. The age range of study subjects used by Gomes *et al.* (28) is higher than 10 years. There is a previous report that subjects aged 5-10 years have more preponderance for the development of subclinical hypothyroidism among children with sickle cell anaemia (9).

The present study shows preponderance of sub-clinical hypothyroidism among children aged 5 – 10 years compared to the older age category, although the statistical analysis did not reach significant level. This finding may be anecdotal because the suggested mechanism in the pathophysiology of hypothyroidism in SCA was tissue ischemia and it will have been assumed that older patients would be more adversely affected because they would have had the disease for a longer period. A similar finding was documented by Kaudha *et al.* (9) among Ugandan children with SCA aged six months to 17 years. This finding suggested that overt hypothyroidism in older age group develops from sub-clinical hypothyroidism, which occurs in the earlier age group. Investigation of this claim was not part of the aims of the current study but merits future study to determine if these three subjects with sub-clinical hypothyroidism eventually develop overt hypothyroidism.

A comment on the effect of gender on the prevalence of sub-clinical hypothyroidism is desirable at this point. It was observed that sub-clinical hypothyroidism was more prevalent among males, subjects with wasting and subjects with severe anaemia. The number of subjects with sub-clinical hypothyroidism is, however, too small to conclude that gender has an effect on the burden of sub-clinical hypothyroidism among children with sickle cell anaemia. This further emphasizes the need for a larger, collaborative study in order to clarify the situation.

When T3, T4 and TSH levels among study subjects were compared with normal reference range, it was observed that no subjects had low TSH. A similar observation was reported in a study conducted by Phuljhele *et al.* (29) among Indian adolescents with sickle cell disease. The current study reported a higher proportion of subjects with high TSH and high T3 than the reported proportion by Phuljhele *et al.* (29) among their studied subjects. For the low T3, Phuljhele *et al.* (29) reported no subjects with either low or high T4 while the present study had proportion of 11.3% each with either low or high T4. The observed disparity may be attributed to environmental factors such as lifestyle, diet and exercise, which influence thyroid hormones levels (24) that are incomparable across both studies.

The present study has demonstrated that children with sickle cell anaemia in our setting are vulnerable to develop sub-clinical hypothyroidism especially at an early age. Routine screening for hypothyroidism is advocated in all children with sickle cell anaemia. It is hoped that the data generated will serve as a reference point for advocacy for routine screening and treatment of hypothyroidism in these group of children in our setting. The limitations of this study include its small sample size and lack of control subjects for comparison. However, the findings of this study buttressed the fact that it is crucial to perform periodic thyroid function evaluations in children with sickle cell anaemia, so as to detect those with sub-clinical hypothyroidism early and apply measures which can delay the progression to clinical hypothyroidism.
